# Identification of inhibitors of ovarian cancer stem-like cells by high-throughput screening

**DOI:** 10.1186/1757-2215-5-30

**Published:** 2012-10-18

**Authors:** Roman Mezencev, Lijuan Wang, John F McDonald

**Affiliations:** 1 School of Biology and Integrated Cancer Research Center, Georgia Institute of Technology, 310 Ferst Dr, Atlanta, GA, 30332, USA

**Keywords:** High-throughput screening, Ovarian cancer, Cancer stem cells

## Abstract

**Background:**

Ovarian cancer stem cells are characterized by self-renewal capacity, ability to differentiate into distinct lineages, as well as higher invasiveness and resistance to many anticancer agents. Since they may be responsible for the recurrence of ovarian cancer after initial response to chemotherapy, development of new therapies targeting this special cellular subpopulation embedded within bulk ovarian cancers is warranted.

**Methods:**

A high-throughput screening (HTS) campaign was performed with 825 compounds from the Mechanistic Set chemical library [Developmental Therapeutics Program (DTP)/National Cancer Institute (NCI)] against ovarian cancer stem-like cells (CSC) using a resazurin-based cell cytotoxicity assay. Identified sets of active compounds were projected onto self-organizing maps to identify their putative cellular response groups.

**Results:**

From 793 screening compounds with evaluable data, 158 were found to have significant inhibitory effects on ovarian CSC. Computational analysis indicates that the majority of these compounds are associated with mitotic cellular responses.

**Conclusions:**

Our HTS has uncovered a number of candidate compounds that may, after further testing, prove effective in targeting both ovarian CSC and their more differentiated progeny.

## Background

Ovarian cancer is the most lethal of gynecological cancers [1] despite its typically high initial response rate to chemotherapy [2]. Recent evidence supports the existence of ovarian cancer stem-like cells (CSC), characterized by self-renewal capacity, ability to differentiate into distinct lineages, high invasiveness and resistance to a number of anticancer agents [3-6]. Since CSC have been shown to be resistant to most current chemotherapies, the frequent recurrence of ovarian cancer is believed, at least in part, to be attributable to the existence of chemo-resistant sub-populations of cancer cells embedded within bulk tumors [6]. For this reason, there is considerable current interest in the development of new chemotherapies that can effectively target this insidious subpopulation of tumor cells [7].

Thus far, searches for compounds that may be therapeutically effective against CSC have employed two alternative strategies. One approach has been to evaluate molecules known to be inhibitory against pathways believed to be deregulated in CSC (*e.g.*, the Hedgehog, NOTCH, PTEN/AKT and WNT/β-catenin signaling pathways) [8]. This approach has resulted in the identification of several potential therapeutic agents that are currently in clinical trials [9]. A second approach is the high-throughput screening (HTS) of CSC-enriched cell populations with libraries of potential inhibitory compounds. This approach has been productively employed to identify candidate compounds displaying cytotoxic/inhibitory effects on breast cancer [10] and glioma [11,12] CSC.

We have recently reported the isolation and characterization of ovarian CSC from an established ovarian cancer cell line OVCAR-3 [13]. These cells display a variety of features and molecular profiles characteristic of CSC previously isolated from ovarian and other cancer tissues [14-17]. Here we report the results of a high-throughput screening of 825 potential drugs (the National Cancer Institute’s “Mechanistic Set” library) [18,19] against ovarian CSC and the subsequent identification of compounds that display significant potential for future development as ovarian CSC therapeutic agents.

## Methods

### Cells

Spheroids were derived from OVCAR-3 cell line as previously described [13] and grown in the stem cell medium (SCM): DMEM/F12 (1:1) supplemented with 0.4% bovine serum albumin (BSA, Sigma-Aldrich, Inc. St. Louis, MO), 20 ng/mL epidermal growth factor (EGF, Invitrogen Corporation, Carlsbad, CA), 10 ng/mL basic fibroblast growth factor (bFGF, Sigma-Aldrich), 5 μg/mL insulin (Sigma-Aldrich) and 1% antibiotic-antimycotic solution (Mediatech-Cellgro, Manassas, VA) in 100 mm ultra-low attachment Petri dishes (Corning Incorporated, Corning, NY). Spheroids grown under these conditions were dissociated weekly using 0.05% trypsin-0.02% EDTA solution (Lonza, Walkerswille, MD) and sub-cultured until the amount of cells was adequate for HTS.

### Compounds

The NCI Mechanistic Set was provided by the Developmental Therapeutic Program (NCI/NIH) as a set of 825 compounds plated in eleven 96-well plates (plate numbers: 4520–4530; suffix: 69). Basic information on these compounds can be retrieved from the DTP website [20] using plate number as the search parameter. These compounds were selected from 37,836 compounds in the NCI repository to represent a broad range of growth inhibition patterns in the NCI 60 cell line screen [18,19] and consequently, they likely represent a diversity of the modes of action of these compounds. Compounds were supplied by DTP as 1 mM solutions in DMSO.

### HTS and data analysis

Spheroids were dissociated to single cells using trypsin; trypsin was neutralized using Soybean Trypsin Inhibitor (Life Technologies, Grand Island, NY; Catalogue # 17075029), and cells were re-suspended in SCM to a density of 50,000 cells/mL. Cells were plated into flat bottom ultra-low attachment 96 well plates (Corning, Product #3474) in a volume of 198 μL per well (200 μL of SCM for blank wells) and incubated for 24 h at 37°C and humidified atmosphere with 5% CO_2_. Drug dilutions were prepared as follows: the eleven supplied NCI Mechanistic Set plates were copied (4 μL of DMSO solution per well) into sterile, round bottom polypropylene 96-well plates (Corning, Product #3359) and each drug was diluted with 22 μL media (working concentrations 153.8 μM). 3 μL of diluted library were added to 198 μL of cells (4 replicated wells for each drug), which resulted in final drug concentration of 2.29 μM. The plates were incubated for 96 h at 37°C and humidified atmosphere with 5% CO_2_. Thereafter, 20 μL of TOX8 reagent (Sigma-Aldrich) were added to each well and after 4-h incubation fluorescence intensities were measured for each well at 560 nm (excitation) and 590 nm (emission). The resazurin (Alamar blue)-based TOX8 reagent has been previously established as a reliable method for determining cell viability/cytotoxicity of tumor spheroid cell cultures [21,22].

Fluorescence intensities for replicated wells were analyzed using Grubb’s test for detecting outliers at critical Z = 1.48 and outliers were removed from the dataset (test was applied only once for each replicated set of values). Percent growth of treated cells relative to untreated control cells was calculated from fluorescence intensities using the formula: $\%\text{growth =}\;100\times \left[\left(\text{average for treated cells - average for blank wells}\right)\right]/\left[\left(\text{average for untreated cells}\right)-\left(\text{average for blank wells}\right)\right]$

Other parameters recommended by Inglese et al. [23], HTS plate design, assay performance evaluation and systematic error detection are presented in Additional file 1.

Of the 793 compounds that passed our assay performance standard, 99 displayed a single outlier among 4 replicated fluorescence intensity values and these outliers were removed before % growth values were calculated.

Statistical significance of differences between fluorescence intensities of drug-treated and untreated control wells was evaluated using Welch’s *t*-test followed by Holm’s step-down method for multiplicity adjustment [24]. Two-sided p-values were determined by Welch’s *t*-test from raw fluorescence intensities corresponding to replicated treated and untreated control wells. These p-values represent probabilities that the difference between mean signal intensities for treated and control wells were obtained by chance. Holm’s procedure was applied to 793 p-values (compounds that passed assay performance test) to counteract the problem of multiple comparisons and to ascertain that the probability of falsely identifying one or more compounds as significantly affecting growth of ovarian cancer stem-like cells is not more than 10%. Thus, compounds selected by controlling for family-wise error rate (FWER) of 0.10 were classified as compounds with statistically significant effect on growth of ovarian cancer stem-like cells. Drugs with % growth 80-110% were considered as compounds with no effect on growth of ovarian cancer stem-like cells.

Results of HTS were interpreted (i) in the context of the activity of FDA-approved drugs present in the library; (ii) in comparison with the potencies of screened compounds against OVCAR-3 cell line (parental cells from which CSC were isolated), and (iii) via mapping to self-organizing maps (SOMs).

A list of 97 FDA-approved oncology drugs was built from the data on the Approved Oncology Drug Set accessible at the DTP website [25] and made available in Additional file 2.

Potencies of library compounds against OVCAR-3 cell line (Dec 2010 release) were retrieved from the DTP website [26]. If multiple GI_50_ values were available for the same compound, the average was taken without inclusion of default values (Note: NCI does not provide descriptive statistics (SD or SEM) for the determined GI_50_ values for tested cells).

All 793 library compounds that passed our assay performance criteria and selected subsets of compounds active against CSC were mapped onto SOMs using the web-based tool 3D MIND developed by the Covell group at the National Cancer Institute [27]. The SOM method represents a type of artificial neural network trained using unsupervised learning to cluster high dimensional data and project them into a low dimensional space. SOMs used in this work was generated from GI_50_ values for ~30,000 compounds across 60 cell lines and consists of 1,350 hexagonal clusters with 9 major cellular response categories: mitosis (M), membrane function (N), nucleic acid metabolism (S), metabolic stress and cell survival (Q), kinases/phosphatases and oxidative stress (P) and 4 unexplored regions (RFJV) [28]. The significance of differences in the distribution of CSC active subsets and all library compounds to these areas was evaluated using Fisher’s exact test with Yate’s continuity correction and the difference was considered significant for two-sided p-value < 0.01.

### Determination of GI_50_

GI_50_ values (concentrations of tested agents that inhibited growth of CSC cell cultures after 96-h incubation to 50% of the untreated control) were determined for 5 compounds (NSC72961, NSC302979, NSC82116, NSC673622 and NSC243928) randomly selected from those resulting in ≤ 50% cellular growth relative to untreated controls. GI_50_ values were determined from concentration-response data generated by the same assay and cell system as used in our HTS. For each compound, 5 concentrations were used (15.6 nM, 62.5 nM, 250 nM, 1000 nM and 4,000 nM). Percent growth (control based normalization) was calculated as described in (Additional file 1: Table S1) and GI_50_ values were determined by non-linear regression of log-transformed data using a normalized response-variable slope model (GraphPad Prism 5.01; GraphPad Software, Inc.) and expressed as mean ± SEM.

## Results and discussion

### HTS identified over 100 compounds that significantly inhibit ovarian CSC growth

NCI’s Developmental Therapeutics Program (DTP) maintains a repository of synthetic and naturally occurring potential anticancer drugs. A sub-set of these compounds, termed the “Mechanistic Set”, represents the range of distinct growth inhibition patterns observed when the repository compounds were tested against the NCI-60 panel of cancer cell lines [18,19].

The CSC culture used in this study was derived from OVCAR-3 cells and is composed of tumor spheroids enriched with slowly proliferating self renewing stem-like cells that have been previously demonstrated to resist apoptosis after detachment from the surface (anoikis), a property known to be prerequisite for invasion and metastasis [13]. In addition, these cells have been shown to display significantly higher invasiveness, migration potential, and resistance to standard anticancer agents relative to the parental OVCAR-3 cells, as well as an ability to differentiate from a CD44-positive/mesenchymal-like phenotype to CD44-negative/epithelial like phenotype [13].

CSC were seeded in 96-well plates and allowed to proliferate for 24 h prior to exposure to the 825 compounds comprising the NCI Mechanistic Set of experimental compounds (chemical library). After 96 h of exposure, % growth of treated cells relative to untreated controls was determined. To reduce the possibility of spurious results, we conducted an assay performance test and subsequently excluded 32 compounds from our survey that were associated with unreliable results (Additional file 3). Of the remaining 793 compounds (Additional file 4), 329 (41.5%) did not display an appreciable effect on ovarian CSC growth (80-110% growth), while 161 compounds (20.3%) displayed a statistically significant effect at FWER=0.10 with 158 compounds displaying an inhibiting effect (3.3-74.1% growth) and 3 compounds displaying a stimulatory effect (135.6-158.7% growth).

In order to focus on the most inhibitory compounds, we operationally defined CSC inhibitory compounds as those displaying a ≤ 50 % cell growth relative to untreated controls. Based on these criteria, 136 of the 793 compounds (17.2%) were classified as inhibitory (range of inhibitory growth: 3.3-50.4%) (Table 1). These 136 inhibitory compounds are listed in Table 1 by a NCI compound number (NSC). A full list the various names associated with each NCI compound number is available as an ASCII file at the National Cancer Institute’s Developmental Therapeutics Program website [29].

**Table 1:** **List of 136 CSC-inhibitory compounds identified by HTS (%growth ≤ 50%, p-adj. ≤ 0.1) compared to GI**_**50 **_**of OVCAR-3 cells**

**NSC**	**%growth**	**SD**	**p-value**	**p-adj (Holms)**	**GI**_**50**_**(-log 10) OVCAR-3**
618332	3.3	4.0	3.73E-07	2.78E-04	5.08
219734	4.2	2.0	9.47E-07	6.88E-04	6.43
128305	4.9	3.4	8.02E-06	5.59E-03	4.34
168597	5.1	5.5	2.42E-06	1.73E-03	7.69
328426	5.3	3.2	4.64E-07	3.44E-04	7.62
143648	5.6	2.3	1.64E-07	1.23E-04	7.43
165563	6.1	4.4	3.58E-07	2.67E-04	8.05
145366	7.1	2.1	5.21E-08	4.00E-05	5.75
636132	7.3	3.2	9.47E-07	6.89E-04	4.00
323241	7.7	2.4	1.59E-07	1.20E-04	7.54
622732	8.0	4.9	4.93E-06	3.46E-03	4.90
208913	8.0	3.5	8.17E-07	5.96E-04	3.80
306864	8.0	3.1	3.86E-07	2.88E-04	5.76
4320	8.0	3.3	6.94E-08	5.31E-05	9.56
3053	8.4	3.9	1.38E-08	1.07E-05	8.54
265450	8.5	3.2	8.14E-08	6.21E-05	7.20
354844	8.6	6.0	4.34E-07	3.22E-04	7.26
164914	8.8	3.7	2.95E-09	2.32E-06	6.75
63701	8.9	3.4	1.00E-09	7.88E-07	7.46
697726	9.0	3.5	5.32E-09	4.16E-06	7.56
637578	9.1	2.3	1.04E-07	7.87E-05	8.36
614928	9.3	2.0	7.60E-07	5.56E-04	4.51
667467	9.8	3.9	3.53E-10	2.79E-07	4.00
65937	10.2	2.1	1.08E-07	8.16E-05	-
65423	10.4	3.1	2.61E-10	2.06E-07	5.78
690634	10.8	2.9	7.52E-07	5.51E-04	7.59
24559	10.8	3.6	9.91E-11	7.86E-08	7.35
18268	10.9	3.4	2.24E-08	1.73E-05	8.41
243023	11.0	3.4	4.48E-06	3.15E-03	8.07
635448	11.1	5.9	1.19E-08	9.27E-06	7.69
7525	11.3	2.4	4.99E-06	3.49E-03	7.60
616232	11.6	4.0	3.97E-10	3.13E-07	4.00
269754	11.9	8.4	1.62E-04	1.03E-01	8.10
680506	12.4	5.0	4.85E-07	3.58E-04	-
58514	12.5	3.1	1.34E-08	1.04E-05	9.52
353527	12.8	4.1	7.25E-07	5.32E-04	7.77
106408	12.8	2.4	1.29E-06	9.30E-04	7.34
620358	13.0	5.5	1.37E-05	9.27E-03	5.72
65104	13.2	4.3	9.57E-07	6.95E-04	8.76
325319	13.2	2.7	7.94E-07	5.80E-04	8.23
328166	13.4	5.4	4.72E-07	3.49E-04	7.35
658144	13.4	2.6	1.51E-08	1.17E-05	6.68
260610	13.9	5.5	2.48E-06	1.77E-03	6.79
631529	14.5	3.2	4.08E-07	3.04E-04	5.82
85236	14.5	2.5	8.67E-08	6.61E-05	5.82
376265	14.7	7.5	1.41E-05	9.53E-03	8.50
349644	14.9	4.6	2.95E-05	1.95E-02	7.72
89671	15.0	2.5	6.73E-07	4.95E-04	7.55
105808	15.3	3.8	1.15E-05	7.87E-03	5.96
635121	16.0	5.3	9.44E-06	6.53E-03	5.52
93419	17.1	7.4	2.92E-05	1.93E-02	6.13
268251	17.4	6.2	2.99E-06	2.12E-03	8.96
202000	17.7	5.0	3.03E-09	2.38E-06	4.05
659999	18.0	6.4	1.30E-08	1.01E-05	5.99
172924	18.5	3.1	7.81E-08	5.97E-05	6.67
673622	18.5	9.1	1.83E-05	1.23E-02	6.59
146604	19.2	4.6	2.41E-09	1.90E-06	6.65
349156	19.5	2.6	1.73E-06	1.24E-03	6.92
526417	19.7	3.8	5.38E-08	4.13E-05	9.28
400978	20.3	2.4	7.26E-09	5.67E-06	7.97
24817	20.4	5.8	2.23E-08	1.72E-05	7.67
319726	20.6	4.0	5.30E-09	4.15E-06	8.00
614826	20.8	5.2	1.41E-05	9.52E-03	6.66
679524	20.9	3.5	1.94E-10	1.54E-07	7.50
629301	20.9	5.5	2.23E-05	1.49E-02	4.80
129414	21.1	7.4	5.48E-05	3.58E-02	7.41
337766	21.4	5.8	1.20E-08	9.34E-06	6.73
7532	21.7	8.3	3.12E-06	2.21E-03	8.00
172946	21.7	5.6	1.25E-05	8.50E-03	7.46
328587	22.1	4.6	2.91E-09	2.29E-06	5.77
34391	22.2	4.2	4.20E-07	3.12E-04	5.60
153858	22.5	4.8	9.62E-07	6.97E-04	9.48
625483	22.8	9.7	2.53E-05	1.68E-02	4.66
82116	23.0	5.7	1.02E-07	7.75E-05	-
72961	23.1	4.4	1.27E-07	9.59E-05	5.60
65380	23.6	6.9	9.38E-05	6.03E-02	7.20
85700	23.7	4.7	1.18E-08	9.20E-06	5.98
336628	23.7	5.9	2.62E-07	1.97E-04	4.77
345647	23.8	48.5	1.03E-05	7.09E-03	6.59
102811	23.9	5.3	1.99E-08	1.54E-05	6.96
669356	24.5	8.1	2.09E-06	1.49E-03	8.00
7521	24.5	6.9	9.49E-05	6.08E-02	7.52
352876	24.7	6.7	4.35E-05	2.86E-02	6.27
148958	24.9	6.9	1.03E-07	7.81E-05	3.09
613009	25.0	5.7	3.89E-06	2.75E-03	7.41
10447	25.5	6.6	3.49E-07	2.61E-04	4.65
301460	25.6	5.0	2.20E-06	1.57E-03	8.00
605756	25.8	6.4	6.31E-07	4.65E-04	5.68
407806	26.5	3.1	1.95E-06	1.40E-03	7.44
67574	27.0	6.0	1.21E-06	8.74E-04	7.68
700582	27.2	5.8	1.02E-07	7.74E-05	6.57
304421	27.9	4.2	4.98E-06	3.49E-03	6.47
521777	28.1	6.1	3.29E-08	2.53E-05	8.00
330500	29.0	4.8	6.10E-07	4.50E-04	6.05
255109	29.6	4.9	1.15E-05	7.84E-03	7.02
302979	30.4	10.8	5.65E-05	3.68E-02	5.43
33410	30.8	5.6	6.80E-08	5.21E-05	7.98
693632	30.8	7.4	2.11E-07	1.58E-04	6.44
24818	31.0	5.3	9.92E-06	6.85E-03	8.12
24819	31.1	4.7	1.14E-06	8.25E-04	8.47
52141	31.3	6.0	1.14E-06	8.24E-04	7.14
96932	31.4	6.9	6.94E-07	5.10E-04	6.77
349155	31.7	7.1	1.99E-05	1.33E-02	6.45
97911	32.4	7.6	6.23E-05	4.05E-02	5.70
243928	32.5	9.0	8.80E-06	6.11E-03	5.69
83265	34.8	6.7	4.05E-06	2.86E-03	5.70
637993	34.8	9.9	8.30E-05	5.36E-02	5.50
145669	34.9	6.6	3.26E-07	2.44E-04	7.75
157930	34.9	6.5	5.26E-06	3.68E-03	6.16
132791	35.2	15.3	1.52E-04	9.67E-02	6.96
331757	35.6	15.4	1.55E-04	9.84E-02	5.11
269142	36.0	10.3	9.02E-06	6.25E-03	6.68
1906	36.2	3.5	4.46E-08	3.43E-05	4.15
14229	37.6	6.1	9.93E-08	7.56E-05	5.55
1620	37.7	8.4	4.88E-06	3.43E-03	4.00
622627	38.4	11.4	1.82E-05	1.22E-02	5.28
215989	38.6	7.1	2.91E-06	2.07E-03	-
13973	40.2	5.5	1.38E-06	9.94E-04	6.28
332598	40.6	7.4	2.18E-05	1.45E-02	10.38
126727	41.3	11.0	6.20E-05	4.04E-02	-
248436	41.4	9.5	1.35E-05	9.15E-03	4.08
705330	41.6	10.5	1.35E-05	9.17E-03	5.70
166454	41.9	7.2	8.76E-07	6.39E-04	5.41
84074	42.9	7.3	1.84E-05	1.23E-02	5.46
632841	43.2	6.1	1.47E-06	1.06E-03	5.83
116693	43.6	5.1	1.86E-07	1.40E-04	5.50
375575	44.8	5.1	1.04E-05	7.14E-03	5.71
98904	45.5	11.5	1.23E-04	7.85E-02	6.05
629971	46.5	10.7	1.01E-05	6.96E-03	6.54
403883	48.3	7.0	2.37E-05	1.57E-02	4.00
267033	48.5	10.9	4.31E-05	2.84E-02	6.35
36437	48.5	5.5	8.51E-06	5.91E-03	5.23
49842	49.1	10.6	2.34E-05	1.56E-02	9.71
654259	49.8	9.0	2.57E-06	1.83E-03	7.23
182986	50.1	9.1	1.52E-05	1.02E-02	5.27
93739	50.4	6.5	1.54E-05	1.04E-02	5.40

**Figure 1:**
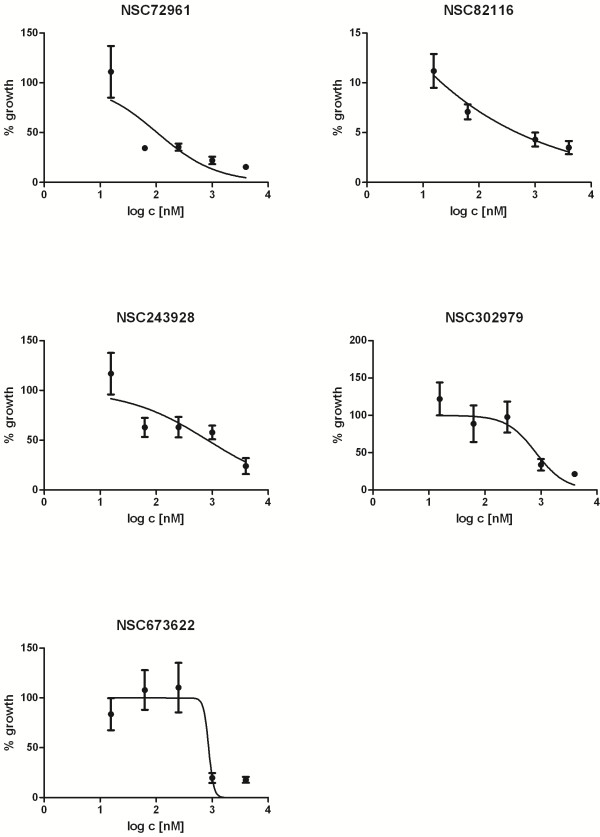
**Concentration**-**response curves for 5 library compounds identified as inhibitors of ovarian cancer stem-like cells.** Curves are fitted by non-linear regression of log-transformed data using a normalized response-variable slope model. Error bars: SEM.

**Figure 2:**
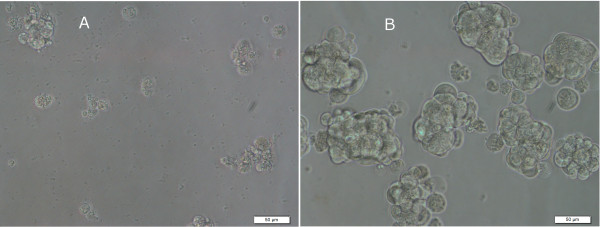
**Phenotypic effect of compound NSC72961 (8-azaadenosine) at 4 μM (96 h) on ovarian cancer stem-like cell culture.** Test treatment (**A**); solvent control (**B**). Scale bar = 50 μm.

We randomly selected 5 of the 136 inhibitory compounds for confirmatory testing. In each case, the determined GI_50_ values were indicative of a significant inhibitory effect: NSC72961: 104.2±13.54 nM; NSC302979: 814.0±84.44 nM; NSC82116: < 1 nM; NSC673622: 865.2 nM (SEM not determined); NSC243928: 831.6±134.72 nM (Figure 1). The cellular phenotype of CSC after treatment with inhibitory compounds is consistent with cell death/apoptosis (Figure 2).

Included among the 793 compounds passing our performance test are 19 drugs previously approved by the FDA for cancer treatment (Table 2). Five of these drugs are among the 136 compounds designated as CSC-inhibitory, but none are commonly used in the treatment of epithelial ovarian cancer. On the other hand, triethylenemelamine (altretamine, NSC9706), which is used for palliative treatment of persistent or recurrent ovarian cancer [30], induced non-significant stimulation of CSC growth in our screening (Table 2).

**Table 2:** Results of HTS of CSC with 19 FDA-approved oncology drugs

**NSC**	**Name**	**% growth**	**SD (%)**	**p-value**	**p-adj**	**GI**_**50**_**OVCAR3 (−log10)**
3053	Dactinomycin	8.4	3.9	1.38E-08	1.07E-05	8.54
24559	Plicamycin	10.8	3.6	9.91E-11	7.86E-08	7.35
67574	Vincristine	27.0	6.0	1.21E-06	8.74E-04	7.68
14229	Mepacrine	37.6	6.1	9.93E-08	7.56E-05	5.55
49842	Vinblastine	49.1	10.6	2.34E-05	1.56E-02	9.71
125066	Bleomycin	53.0	10.2	1.10E-05	7.54E-03	5.28
63878	Cytarabine	76.6	11.0	1.04E-02	1	5.00
105014	Cladribine	82.7	17.0	7.13E-02	1	4.59
755	Mercaptopurine	95.2	8.4	2.83E-01	1	5.94
740	Methotrexate	96.7	30.8	8.38E-01	1	6.69
226080	Rapamycin	97.0	20.9	7.93E-01	1	8.17
296961	Amifostine	99.4	12.0	9.18E-01	1	3.13
85998	Streptozocin	103.7	28.9	8.10E-01	1	3.14
32065	Hydroxyurea	107.4	14.8	2.38E-01	1	2.92
180973	Tamoxifen	115.1	14.3	4.45E-02	1	5.23
750	Busulfan	120.6	15.9	3.65E-02	1	3.60
38721	Mitotane	128.2	47.6	2.68E-01	1	4.73
9706	Triethylenemelamine	128.9	82.5	5.26E-01	1	4.74
45388	Dacarbazine	164.3	14.2	4.46E-04	2.68E-01	4.25

The two most CSC-inhibitory of the FDA-approved drugs, dactinomycin (NSC 3035; 8.4% cell growth) [31] and plicamycin (mithramycin A, NSC 24559; 10.8% cell growth) [32] have both been previously reported to induce programmed cell death or apoptosis by inhibiting RNA transcription. Dactinomycin is used in the treatment of several cancers including gestational trophoblastic neoplasia [33] and Wilms’ tumor [34]. Plicamycin has been used in the treatment of testicular cancer [35] and hypercalcemia associated with advanced malignancy [36]. The anti-microtubule drug vincristine (NSC24559) is used in the treatment of acute leukemias, Hodgkin lymphoma, and aggressive non-Hodgkin lymphoma, but is also included in combinations for treatment of small-cell lung cancer, breast cancers and some pediatric neoplasms [37]. Vinblastine (NSC49842) is used in the treatment of Hodgkin’s lymphoma [37], and in combination with cisplatin and bleomycin in the treatment of testicular and ovarian germ cell cancers [38]. Mepacrine (quinacrine, NSC14229), an inhibitor of NFκB [39] and topoisomerase activity [40], is primarily used as an antimalarial drug [41]. In oncology, it is most commonly used for the treatment of pleural effusions in advanced malignant diseases [42].

### Most ovarian CSC growth-inhibiting compounds also inhibit the growth of more differentiated ovarian cancer cells

It has been established previously that normal stem cells are more resistant to the induction of apoptosis by radiation and cytotoxic agents than their differentiated progeny and similarly, CSC have been shown to display increased resistance to these same agents relative to the more differentiated cells that comprise the bulk of the tumor [43-45]. Indeed, it has been proposed that this dichotomy may contribute to the recurrence of cancer growth after the initial response of tumors to chemotherapeutic treatments [46]. Consistent with this view, we previously reported that several ovarian cancer drugs (*e.g.*, NSC119875-cisplatin, NSC724770-docetaxel, NSC609699-topotecan) that are effective against ovarian cancer OVCAR-3 cells, are significantly less effective at inhibiting growth of ovarian CSC [13]. To assess if the apparent dichotomy in drug effectiveness between ovarian CSC and their more differentiated progeny is characteristic of the 136 ovarian CSC inhibitory compounds identified in our study, we sought to compare the results of our HTS with NCI’s previous testing of compounds against the OVCAR-3 cell line. In the NCI program, GI_50_ values (concentrations required to inhibit growth by 50%) on OVCAR-3 cells were determined for nearly all of the compounds used in our HTS. The OVCAR-3 GI_50_ values (expressed as -log_10_ GI_50_) for 136 of the CSC-inhibitory compounds are presented in Table 1. The data output from our HTS is relative % growth rather than GI_50_ values. However, since the concentration of compounds used in our HTS was 2.29 μM (see Methods), the GI_50_ for compounds resulting in ≤ 50% growth of CSC is predicted to be ≤ 2.29 μM or ≥ 5.64 on the -log_10_ scale (−log_10_ 2.29×10^-6^ = 5.64). By comparing this value with the -log_10_ GI_50_ values previously determined for OVCAR-3 cells in the NCI study, we found that 73% (99/136) of the compounds that we designated as CSC inhibitory compounds are also inhibitory for OVCAR-3 cells (*i.e.*, -log_10_ GI_50_ ≥ 5.64). This suggests that there may be a number of inhibitory compounds with the potential to target both ovarian CSC and their more differentiated progeny. Of the remaining 37 (136–99) CSC-inhibitory compounds, 5 were not previously tested on OVCAR-3. Thus, based on our criteria, we classified 32 compounds to be preferentially inhibitory for CSC (Table 3).

**Table 3:** Compounds preferentially inhibitory for CSC

**NSC**	**Name**	**% growth**	**SD**	**p-adj**
618332	2,3-Dibromonaphthoquinone	3.3	4.0	2.78E-04
128305	5,7-Dihydroxy-3',4'-dimethoxyflavone	4.9	3.4	5.59E-03
636132	3-Cyano-N,3-bis(2-methylphenyl)-2-oxopropanamide	7.3	3.2	6.89E-04
622732	N-(4-chlorophenyl)-1-methyl-1H-pyrazolo[3,4-b]quinolin-5-amine	8.0	4.9	3.46E-03
208913	Ethyl 2-(((1-adamantyl(methyl)amino) carbonyl)amino)propanoate	8.0	3.5	5.96E-04
614928	3,3,4,4-Tetramethyltetrahydro-2,5-furandiol	9.3	2.0	5.56E-04
667467	2-Phenyl-1,4-thiazino[3,2-c]quinoline-3-thione	9.8	3.9	2.79E-07
616232	Dibromodulcitol	11.6	4.0	3.13E-07
635121	N'-(1-(4H-1,4-benzothiazin-2-yl)ethylidene)-2-hydroxybenzohydrazide	16.0	5.3	6.53E-03
202000	(4Z)-4-[(3,4-dichlorophenyl) methylidene]-2-(furan-2-yl)-1,3-oxazol-5-one	17.7	5.0	2.38E-06
629301	3,6-Dihydro-3,6-ethanocyclohepta[cd] [1]benzofuran-10,10,11,11-tetracarbonitrile	20.9	5.5	1.49E-02
34391	Cryptocyanine iodide	22.2	4.2	3.12E-04
625483	1-(2-Chloro-6-fluorophenyl)-1H,3H-Thiazolo(3,4-a)benzimidazole	22.8	9.7	1.68E-02
72961	8-Azaadenosine	23.1	4.4	9.59E-05
336628	Merbarone	23.7	5.9	1.97E-04
148958	Ftorafur	24.9	6.9	7.81E-05
10447	Purpurin	25.5	6.6	2.61E-04
302979	Shikoccin	30.4	10.8	3.68E-02
637993	6H-Imidazo[4,5,1-de]acridin-6-one, 5-[2-(diethylamino) ethylamino]-8-methox`y-1-methyl-, dihydrochloride	34.8	9.9	5.36E-02
331757	2-Anthracenecarboxamide, N-[4-(diethylamino)-1-methylbutyl]-9,10-dihydro-9,10-dioxo-, monohydrochloride	35.6	15.4	9.84E-02
1906	Piperidinium piperidinedithiocarbamate	36.2	3.5	3.43E-05
14229	Mepacrine	37.6	6.1	7.56E-05
1620	4-[[(2-furanyl)methyl]amino]-1H-pyrazolo[3,4-D]pyrimidine	37.7	8.4	3.43E-03
622627	2-(chloromethyl)-1,3-dinitro-5-(trifluoromethyl)benzene	38.4	11.4	1.22E-02
248436	Platinum, dibromo(6-thioguanosine-N7,S6)-, (SP-4-3)-	41.4	9.5	9.15E-03
166454	Decamine	41.9	7.2	6.39E-04
84074	Phosphonium, (3-bromopropyl)triphenyl- bromide	42.9	7.3	1.23E-02
116693	2,3-bis(benzoyloxy)succinic acid compound with 1,4-dimethyl-2-((4-methylphenyl)(phenyl)-l 4-sulfanyl)benzene (1:1)	43.6	5.1	1.40E-04
403883	Cedran-8-ol	48.3	7.0	1.57E-02
36437	Crassin acetate	48.5	5.5	5.91E-03
182986	Diaziquone	50.1	9.1	1.02E-02
93739	Fuchsine	50.4	6.5	1.04E-02

Among the 99 compounds found to be co-inhibitory for CSC and OVAR-3 cells, four have previously been FDA approved for cancer treatment (NSC3053-dactinomycin; NSC24559-plicamycin; NSC49842-vinblastine; NSC67574-vincristine) (Table 2). Only one of the previously approved cancer drugs, the NFκB-inhibitor Mepacrine (NSC14229), is included among those compounds classified as preferentially inhibitory for ovarian CSC (37.6% growth).

### Computational model classifies CSC inhibitory compounds into predicted cellular response groups

Neither the mode of action nor the molecular targets of the 32 compounds classified by us as preferentially inhibitory for CSC have, as yet, been definitively determined. However, there are a variety of computational tools that can be informative in predicting the putative cellular responses to these compounds and in suggesting lines of future investigation. One such tool, developed by the Covell group at NCI [27,28,47], utilizes data from the treatment of 60 representative human cancer cell lines (the NCI-60 panel) [48] with nearly 30,000 compounds including those investigated in our HTS. These data were used to generate a self-organizing map (SOM) that visualizes the position of ~30,000 screened compounds within 9 major cellular response categories: mitosis (M), membrane function (N), nucleic acid metabolism (S), metabolic stress and cell survival (Q), kinases/phosphatases and oxidative stress (P) and 4 unexplored regions (RFJV).

**Figure 3:**
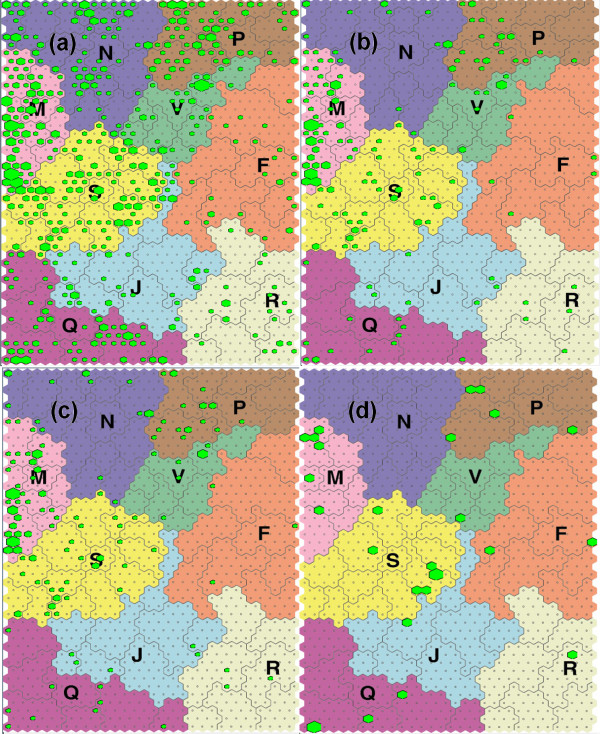
**Mapping of compounds used in the HTS onto SOMs.** (**a**) all 793 evaluable compounds (**b**) 136 CSC inhibitory compounds; (**c**) 99 CSC and OVCAR-3 co-inhibitory compounds; (**d**) 32 CSC-specific inhibitory compounds. [mitosis (M), membrane function (N), nucleic acid metabolism (S), metabolic stress and cell survival (Q), kinases/phosphatases and oxidative stress (P) and 4 unexplored regions (RFJV)].

Using this SOM, we were able to visually compare the predicted cellular responses of all of our screened compounds (that passed assay performance criteria) relative to those identified as having an inhibitory effect on CSC. Shown in Figure 3 is a SOM upon which we have mapped (a) all 793 compounds used in our HTS; (b) the 136 compounds identified as inhibitors of CSC (including compounds that also inhibit OVCAR-3 cells); (c) the 99 compounds identified as co-inhibitory of CSC and OVCAR-3 cells; and (d) the 32 compounds that exert a CSC-specific inhibitory effect.

The results indicate that compared to all 793 evaluable compounds, the 136 identified as inhibitory for CSC are highly significantly enriched for compounds associated with M (mitotic) cellular responses (M: 36.1% vs 18.7%; p<0.0001; Figure 3b and Table 4). This region of SOM contains many compounds known to interfere with microtubule and/or actin filaments, such as taxanes, derivatives of colchicine, vinca alkaloids, rhizoxin and nocodazole [47]. This region also contains compounds associated with inhibition of the DNA polymerase pathway and is associated with the Gene Ontology (GO) terms: Mitotic checkpoint, Cytokinesis, DNA topological change, Cell cycle (Biological Processes); Nucleus, Kinetochore (Cellular Components), and DNA topoisomerase activity (Molecular Function) [28].

**Table 4:** Number of compounds mapping into individual cellular response categories of SOM

	**F**	**J**	**M**	**N**	**P**	**Q**	**R**	**S**	**V**
**(a)**	29 (4.2%)	44 (6.4%)	129 (18.7%)	102 (14.8%)	92 (13.3%)	83 (12.0%)	25 (3.6%)	140 (20.3%)	46 (6.7%)
**(b)**	5 (2.6%)	7 (3.6%)	70 (36.1%)	16 (8.2%)	25 (12.9%)	13 (6.7%)	5 (2.6%)	42 (21.6%)	11 (5.7%)
**(c)**	3 (1.9%)	5 (3.1%)	62 (38.5%)	16 (9.9%)	19 (11.8%)	9 (5.6%)	3 (1.9%)	35 (21.7%)	9 (5.6%)
**(d)**	2 (7.4%)	1 (3.7%)	6 (22.2%)	0 (0%)	5 (18.5%)	4 (14.8%)	1 (3.7%)	7 (25.9%)	1 (3.7%)
**p-ab**	0.3988	0.1653	<0.0001	0.0169	1.0000	0.0365	0.6537	0.6882	0.7412
**p-ac**	0.2469	0.1326	<0.0001	0.1283	0.6970	0.0163	0.3325	0.6663	0.7235
**p-ad**	Numbers too small for statistical evaluation								

(a) all 793 evaluable compounds (b) 136 CSC inhibitory compounds; (c) 99 CSC and OVCAR-3 co-inhibitory compounds; (d) 32 CSC-specific inhibitory compounds. P-values: Fisher’s exact test for the significance of differences between proportions of compounds in a given cellular response category in 793 compound set (A) and compound subset B (p-AB), C (p-AC), or D (p-AD). [mitosis (M), membrane function (N), nucleic acid metabolism (S), metabolic stress and cell survival (Q), kinases/phosphatases and oxidative stress (P) and 4 unexplored regions (RFJV)].

The 99 compounds that were co-inhibitory for OVCAR-3 and CSC also displayed a significant enrichment for M cellular responses (M: 38.5% vs 18.7%; p<0.0001; Figure 3c and Table 4). When the 32 compounds classified as preferentially inhibitory for CSC were mapped, the enrichment for M (mitotic) cellular responses was no longer apparent (Figure 3d). Although this may be attributable to the fact that CSC are less mitotically active than cancer epithelial cells [43], the relatively small number of compounds (32) in this category precludes definitive conclusions.

## Conclusion

We tested the inhibitory effect of 825 compounds (NCI Mechanistic Set) on the growth of ovarian CSC derived from a previously established epithelial ovarian cancer cell line (OVCAR-3) [13]. 158 of these compounds were found to have a significant inhibitory effect on ovarian CSC growth. The most inhibitory of these compounds (≤ 50% growth relative to controls) were designated as CSC inhibitory. Among these 136 CSC inhibitory compounds are 5 FDA-approved cancer drugs, but none of these are commonly used in the treatment of epithelial ovarian cancer. A comparison of the ovarian CSC inhibitory compounds identified in this study with compounds previously shown to be inhibitory for OVCAR-3 ovarian cancer cells revealed an unexpected 73% overlap. Computational analysis indicates that the majority of these compounds are associated with mitotic cellular responses.

While epithelial ovarian cancer is frequently responsive to current chemotherapeutic treatments, disease recurrence remains a persistent problem that has been, at least partially, attributed to the fact that ovarian CSC are resistant to standard therapies [6,13]. Our HTS has uncovered a number of candidate compounds that may, after further testing, prove effective in targeting both ovarian CSC and their more differentiated progeny.

## Abbreviations

CSC: Cancer Stem Cells; DTP: Developmental Therapeutics Program; FDA: U.S. Food and Drug Administration; HTS: High Throughput Screening; NCI: National Cancer Institute; NSC: National Service Center; SOM: Self-Organizing Map.

## Competing interests

All authors declare that they have no competing interests.

## Authors’ contributions

RM and JM conceived the study and wrote the manuscript. RM and LW performed the HTS experiment. RM processed and analyzed the data. All authors read and approved the final manuscript.

## Supplementary Material

Additional file 1 Plate design, assay performance evaluation and systematic error detection doc. Click here for file

Additional file 2 97 drugs from the FDA-approved oncology drug set, Description: NSC – NSC identifier; CAS – CAS identifier. Click here for file

Additional file 3 **Compounds from the 2 plates failing the assay performance test, Description: AP - Assay plate #; NSC – NSC identifier; %growth; SD – standard deviation of %growth; p-value – determined by Welch’s*t*-test between treated and control wells.** Click here for file

Additional file 4 **Results of HTS for 793 compounds that passed the assay performance test (also shown are GI50 values (−log10) for OVCAR-3 cells), Description: AP - Assay Plate; NSC – NSC identifier; %growth; SD – standard deviation of %growth; p-value – determined by Welch’s*t*-test between treated and control wells; rank – rank of compound according to p-value; p-adj_Holms - Holm's procedure-adjusted p-values; position – position of drug in assay plate; OVCAR-3 - GI50 values for OVCAR-3 cell line (retrieved from DTP/NCI).** Click here for file
