# Supplementary material for: Identification of inhibitors of ovarian cancer stem-like cells by high-throughput screening
Source: J Ovarian Res. 2012 Oct 18;5:30. doi: 10.1186/1757-2215-5-30 (PMC3484114; doi:10.1186/1757-2215-5-30)
Supplement: Additional file 1 — Plate design, assay performance evaluation and systematic error detection doc. [file 1757-2215-5-30-S1.doc]

**HTS plate design, assay performance evaluation and systematic error detection**

*Plate Design*

The plate design for HTS is represented in Figure S1. Columns represented in green contained untreated control cells. Columns represented in blue contained cells treated with drugs. Two drugs (X 4 replicates per drug) were tested per column (columns 2,4,5,6,8,9,10 and 11). Colum 1 (yellow) blanks containing no cells or fluid. Column 12 (white) contained only PBS (phosphate buffered saline). Column 3 (A3-H3) contained the untreated controls for the 8 drugs (X 4 replicates per drug) distributed in columns 2, 4, 5 and 6; column 7 (A7-H7) contained the untreated controls for the 8 drugs distributed in columns 8, 9, 10 and 11.


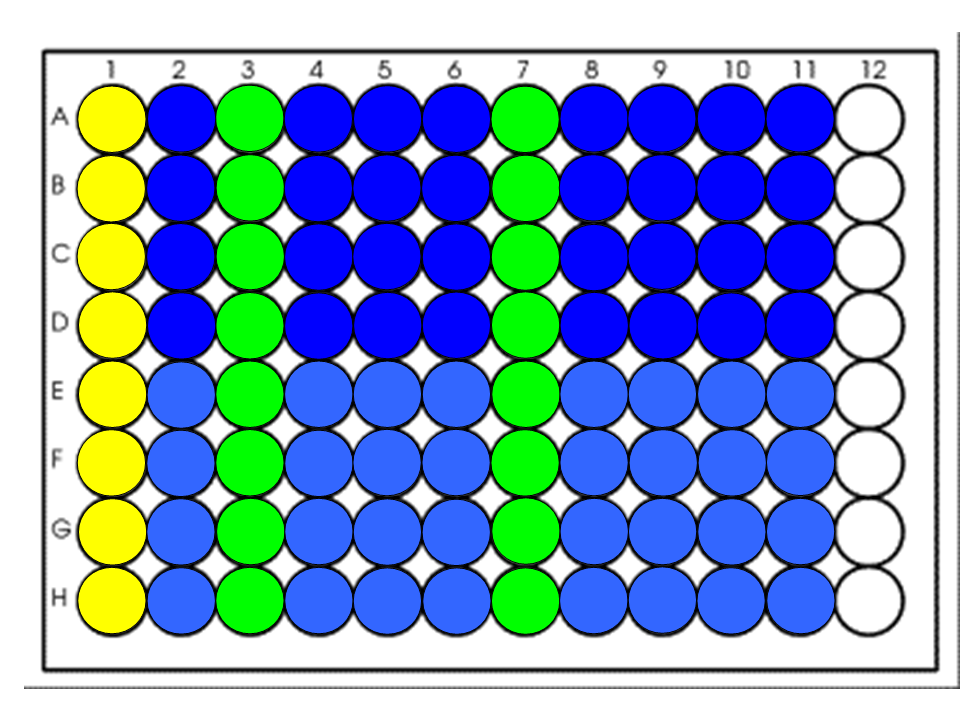


Figure S1 HTS plate design. Each 96 well plate contained 16 drugs in 4 replicated wells for each drug (blue wells). A1-H1: blank wells (yellow); (A3-H3)+(A7-H7): untreated control wells (green). Wells A12-H12 contained only PBS (phosphate buffered saline) and were not used in HTS (white).

Assay performance was estimated using signal-to-background ratio, *i.e*., the ratio of average fluorescence intensity of negative control wells (A3-H3 and separately A7-H7) to average fluorescence intensity of blank wells (A1-H1). Plates for which both these ratios F(A3-H3)/F(A1-H1) and F(A7-H7/A1-H1) were ≤2.0 (low gain of fluorescence in untreated cell wells) were labeled as “assay invalid” and compounds tested in these plates were disregarded. Only two of the 52 plates tested (representing 32 of 825 or 3.9% of compounds tested, see also Additional Material file 3) failed this assay performance standard. These two plates displayed unacceptable signal-to-background ratios of 1.41 and 1.27 for one plate and 1.36 and 1.29 for the other plate.

Parameters describing in detail our HTS experiment are listed in Table S1.

**Table S**1 HTS parameters

| **Category** | **Parameters** | **Details** |
| --- | --- | --- |
| Assay | Nature of the assay | Homogeneous, end-point fluorescence assay |
|  | Assay strategy | Measurement of fluorescence intensity of resorufin generated from resazurin by viable cells treated and untreated (control) with screened agents |
| Library screened | Nature of the library | Compounds selected to represent diversity of response patterns on 60 cell lines for 37,860 compounds from DTP NCI repository |
|  | Details | Molecular structures available in MDL SD file formats:  2D (http://dtpsearch.ncifcrf.gov/MECHSET2D.SDF)  3D (http://dtpsearch.ncifcrf.gov/MECHSET3D.SDF) |
|  | Quality control | All compounds assured by supplier; QC not performed |
|  | Final concentration | 2.29 µM |
| HTS process | Plate controls | Negative control: media without addition of drugs; Positive control: internal (active compounds from library) |
|  | Plate number | 52 plates [16 drugs (4 replicates/drug), 8 blank wells A1-H1 and 16 control wells (A3-H3)+(A7-H7) per plate] |
|  | Reagent and compound dispensing system | Drugs and TOX-8 reagent delivered using epMotion 5070 liquid handling workstation (Eppendorf, Hamburg, FRG) |
|  | Output, detector, analysis SW | Plates read with Synergy 4 Hybrid Multi-Mode Microplate Reader controlled with Gen5 SW (BioTek, Winooski, VT). Fluorescence intensities detected in monochromator mode for λ(exc.) = 560 nm / λ(em.) = 590 nm. Raw data were imported to and processed by Excel. |
|  | Normalization | Control based normalization:  % growth = 100 × [(average for treated cells – average for blank wells)] / [(average for untreated cells) – (average for blank wells)] |
|  | Correction factors | None (systematic error was not detected) |
|  | Performance | Expressed as signal-to-background ratio, *i.e.* the ratio of average fluorescence intensity of negative control wells (A3-H3 and separately A7-H7) to average fluorescence intensity of blank wells (A1-H1). Assay labeled as “invalid” for plates for which F(A3-H3)/F(A1-H1) and F(A7-H7/A1-H1) were ≤2.0 (low gain of fluorescence in untreated cell wells). |

*Systematic error detection*

Systematic error may be caused by various factors such as liquid handling anomalies, reader effects, position-dependent differences in media evaporation from plate wells and its detection was performed using the χ^2^ goodness-of-fit test on the hit distribution surface.^¥1, ¥2^ In this test, plates were divided into 16 sections (A2-D2, E2-H2, A4-D4, E4-H4, A5-D5, E5-H5, A6-D6, E6-H6, A8-D8, E8-H8, A9-D9, E9-H9, A10-D10, E10-H10, A11-D11, E11-H11), each section corresponding to 4 replicated wells for testing of individual library compounds. For systematic error detection, 46 of the plates passing our assay performance test (732 compounds) were used to determine hit distribution across these sections. Hits were selected for the purpose of detection of systematic error as subset of 732 compounds with % growth < average % growth – SD for all 732 compounds. The null hypothesis H_0_ was that the hits are evenly distributed across plate sections (no systematic error present in the data) and test statistic χ^2^ was calculated over all 16 plate sections of the given hit distribution surface as follows:

$$\chi^{2}=\sum_{i=1}^{16} \frac{(x_{i}-E)^{2}}{E}$$

where x_i_ corresponds to the number of hits per section I, and E is total number of hits of the whole hit distribution surface divided by 16 (number of sections) and number of degrees of freedom is 15 (number of sections – 1).

46 plates used for the detection of systematic error included 732 compounds with an overall average % growth of 83.40% (SD=37.68%). 136 compounds with % growth < 45.7% were labeled as hits for the purpose of systematic error detection and the plot of their distribution across 16 plate sections is presented in the Figure S2. The χ^2^-contingency test failed to reject null hypothesis at α=0.05 consistent with the hypothesis that the hit distribution surface is constant (E=8.5 (136/16); χ^2^=19.059; 15 degrees of freedom). The results indicate that there was no systematic error associated with our results.


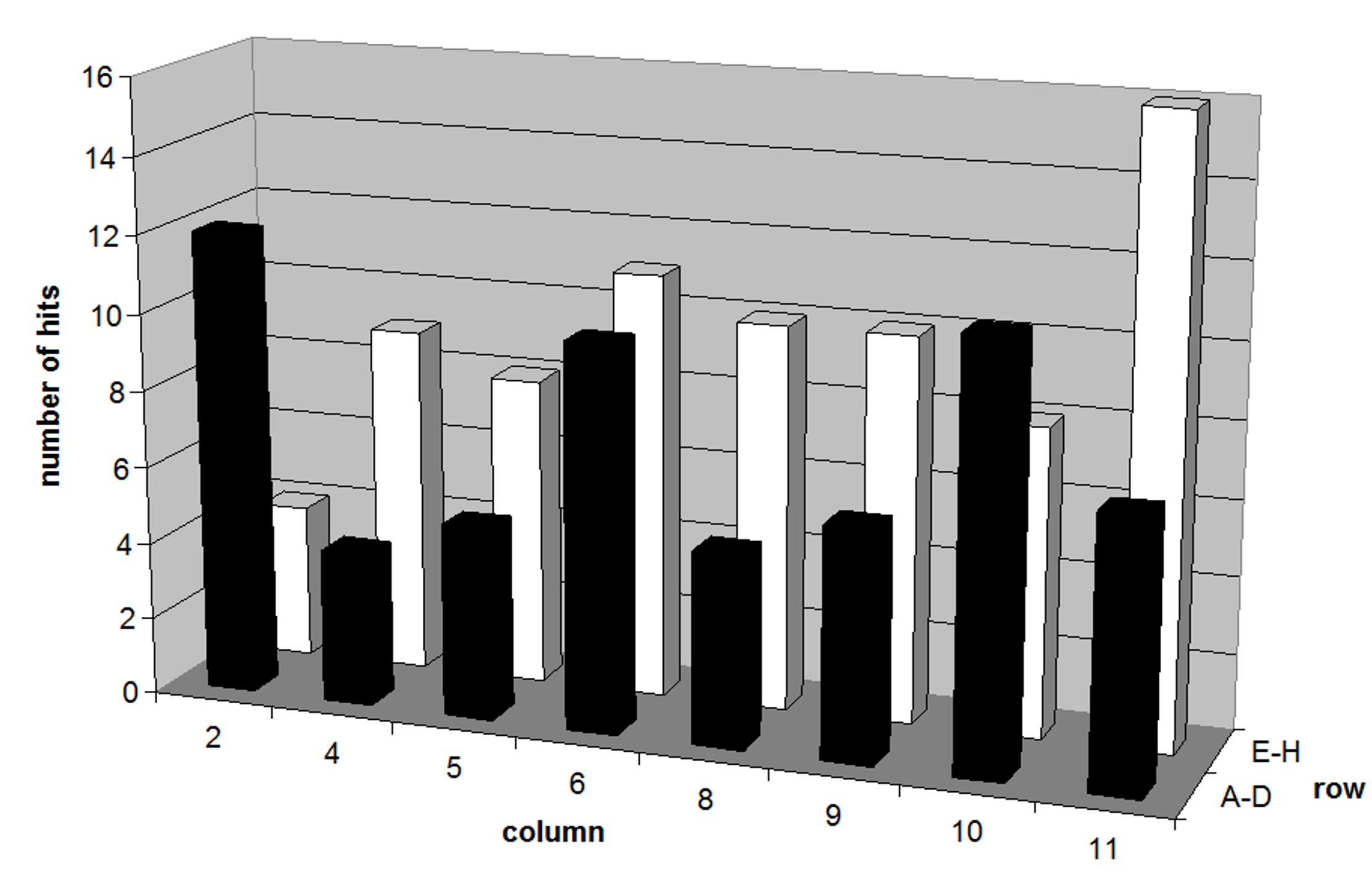


Figure S2 Distribution of hits for detection of systematic error across 16 plate sections used

for drug tests (4 wells per compound). Plate sections are defined by rows and columns (*e.g.* row A-D and column 2 defines plate section consisting of wells A2, B2, C2, and D2). Hits are selected based on X-SD threshold, where X is average % growth (83.40%) and SD is standard deviation of % growth (37.68%) across all 16 sections for 46 plates.

^¥1^ Makarenkov V, Zentilli P, Kevorkov D, Gagarin A, Malo N, Nadon R: **An efficient method for the detection and elimination of systematic error in high-throughput screening.** *Bioinformatics* 2007, **23**:1648-1657.

^¥2^ Dragiev P, Nadon R, Makarenkov V: **Systematic error detection in experimental high-throughput screening.** *BMC Bioinformatics* 2011, **12**:25.
